# Protoparvovirus Knocking at the Nuclear Door

**DOI:** 10.3390/v9100286

**Published:** 2017-10-02

**Authors:** Elina Mäntylä, Michael Kann, Maija Vihinen-Ranta

**Affiliations:** 1 Department of Biological and Environmental Science and Nanoscience Center, University of Jyvaskyla, FI-40500 Jyvaskyla, Finland; elina.h.mantyla@jyu.fi; 2 Laboratoire de Microbiologie Fondamentale et Pathogénicité, University of Bordeaux, UMR 5234, F-33076 Bordeaux, France; michael.kann@u-bordeaux.fr; 3 Centre national de la recherche scientifique (CNRS), Microbiologie Fondamentale et Pathogénicité, UMR 5234, F-33076 Bordeaux, France; 4 Centre Hospitalier Universitaire de Bordeaux, Service de Virologie, F-33076 Bordeaux, France

**Keywords:** protoparvovirus, intracellular dynamics, entry, nuclear import, nuclear envelope, nuclear pore complex, importins, nuclear localization sequence, nuclear envelope break down

## Abstract

Protoparvoviruses target the nucleus due to their dependence on the cellular reproduction machinery during the replication and expression of their single-stranded DNA genome. In recent years, our understanding of the multistep process of the capsid nuclear import has improved, and led to the discovery of unique viral nuclear entry strategies. Preceded by endosomal transport, endosomal escape and microtubule-mediated movement to the vicinity of the nuclear envelope, the protoparvoviruses interact with the nuclear pore complexes. The capsids are transported actively across the nuclear pore complexes using nuclear import receptors. The nuclear import is sometimes accompanied by structural changes in the nuclear envelope, and is completed by intranuclear disassembly of capsids and chromatinization of the viral genome. This review discusses the nuclear import strategies of protoparvoviruses and describes its dynamics comprising active and passive movement, and directed and diffusive motion of capsids in the molecularly crowded environment of the cell.

## 1. Introduction

DNA viruses (with exception of *poxviridae* family), and some RNA viruses, such as retroviruses, orthomyxoviruses and bornaviruses, have to enter the cell nucleus due to their need of the nuclear DNA replication and transcription machinery. Their genomes have to be transported from the cell periphery into the nucleus facilitated by multiple coordinated interactions between the virus (or a subviral structure) and host proteins. Further, the viral genome has to be released at a defined site in order to allow replication. Growing evidence shows that, for most viruses, uncoating occurs at the cytoplasmic side of the nuclear envelope (NE).

The genus protoparvovirus in the subfamily *Parvovirinae* includes naked single-stranded DNA viruses with a wide host spectrum from carnivores and primates to rodents [1]. Among carnivores they infect various species including dogs (canine parvovirus (CPV)), minks (mink enteritis virus (MEV)) and cats (feline parvovirus (FPV)). Primate protoparvoviruses infect humans (bufavirus, tusavirus, cutavirus), rodent protoparvoviruses infect mice (minute virus of mice (MVM)) and rats (parvovirus H-1 (PV H-1), rat parvovirus 1 (RPV1)), and ungulate protoparvoviruses infect swines (porcine parvovirus (PPV)). While some protoparvoviruses cause diseases such as MVM, CPV and FPV, others are not associated with any illness, e.g., bufavirus and PV H-1. Research on PVs is not only driven by disease prevention and cure but also by their use in parvovirus-based virotherapy. Some protoparvoviruses like PV H-1 possess natural oncotropism and their cytotoxicity is used in experimental cancer therapy [2,3].

Members of protoparvoviruses have a small, icosahedral and a non-enveloped capsid of 18–26 nm in diameter. The capsid encloses a single-stranded, linear, non-segmented and often negative-sense DNA genome of 4–6 kb in length with duplex hairpin telomeres at both termini [4,5]. Although rare, certain protoparvovirus species (e.g., LuIII) also encapsidate positive strands [6]. While the genome has two genes transcribed from two open reading frames (ORFs) [7,8], alternative splicing and proteolytic cleavage account for synthesis of 4 to 5 proteins. The left-hand ORF encodes two non-structural (NS1-2) proteins, both having multiple functions in the viral life cycle. NS1 regulates viral gene expression [9], and induces cell cycle arrest and apoptosis [10,11]. NS1 is also involved in the induction of single-stranded DNA breaks, blockage of cellular DNA replication [12] and alteration of cytoskeletal and the nuclear structures [13,14]. In virions, the protein is covalently bound to the 5’ end of the viral DNA [15]. Exposed on the virus surface, it becomes removed during cell entry upon new infection [16]. NS2 plays an indirect role in parvovirus infection facilitating viral replication and capsid assembly. NS2 interactions are not well understood but it has a critical role in active nuclear export of mature particles via binding to the nuclear export factor Crm1 and modulation of viral host range [17,18,19,20].

The right-hand ORF encodes three structural viral proteins (VPs 1–3). The capsid has a T = 1 symmetry formed from 60 copies of the VPs, which are splice variants of the VP1 gene [21]. The largest protein, VP1 constitutes ~10% of the capsid proteins. The major capsid protein is VP2, which is involved in the nuclear assembly of capsids [22,23]. The VP1 and VP2 have a common C-terminal sequence and the complete sequence of VP2 (64 to 66 kD) is contained in VP1 (83 to 86 kD) [5,24]. In addition, the VP1 has a unique N-terminal domain (VP1u). VP3 is the smallest capsid protein formed by a 15 to 20 aa cleavage from the N-terminus of VP2. It is only found in mature DNA-containing capsids, where its ratio with VP2 defines the virus species and maturity [25,26]. Because of their high immunogenicity, the VPs are the main antigens of these viruses [27,28].

Parvoviruses exploit multiple cellular processes including endocytic pathways, and active nuclear transport during the initial phase of infection. Capsids enter the cell via receptor-mediated endocytosis for genome transport toward the nucleus. Transport to the nuclear periphery is followed by endosomal release of the capsids into the cytoplasm [29,30,31,32,33,34]. Nuclear import of the genome is dependent on capsid interactions with the nuclear transport machinery, including members of the karyopherin β superfamily (importins) and nuclear pore complexes (NPCs). Nuclear import of capsids and their disassembly are followed by genome replication and viral assembly, which require the import of structural and nonstructural viral proteins.

## 2. Cytoplasmic Transport

At early stages of the endocytic pathway, vesicles from the plasma membrane are rapidly targeted to the early endosomes. Maturation of early-to-late endosomes with an acidic pH of 4.5–5.5 is followed by the formation of enlarged multivesicular structures [35]. The fusion of an endosome with a lysosome generates a transient hybrid organelle, the endolysosome [36]. The endolysosomes are converted to classical dense lysosomes, which constitute a storage organelle for lysosomal hydrolases used for degradation of macromolecules [37]. Evidently, viruses using this entry route had to develop a strategy for endosomal escape prior to lysosomal degradation, with the exception of reoviruses, which require lysosomal proteases for their partial uncoating [38].

After the stepwise endosomal uptake, CPV and other parvoviruses, including adeno-associated virus 2 (AAV2), a member of the genus *dependoparvovirus,* are present in small vesicles scattered around the cytoplasm, and in large vesicles accumulated in the perinuclear area [30,39,40,41,42,43]. This is in line with earlier observations of size-distribution of cargo-filled endosomes and their perinuclear accumulation: small endosomes with little cargo are found in the cell periphery, whereas large endosomes rich in cargo are located close to the nucleus [44,45,46]. The endosome-associated movement of viral capsids fluctuates from fast to slow. The fast movement could correspond to capsids located in small endosomes actively moving along microtubules (MTs) [43]. The slow movement corresponds to capsids accumulated in endosomes at the perinuclear area, where they move very slowly or are nearly immobile (velocity < 1 µm/s) [43,45,46,47,48,49,50,51]. In parvovirus entry, the endosomal transportation is followed by capsid release into cytoplasm, which appears to be inefficient. This is induced by the low endosomal pH leading to activation of a phospholipase A2 (PLA2) activity located in the N terminus of VP1 [52].

The high viscosity and molecular crowding restricts viral capsid motility in the cytoplasm requiring active and directed transport to the nucleus. Active transport can be facilitated by actin polymerization as observed for intracellular vaccinia- and baculoviruses [53,54]. However, nearly all viruses with a nuclear phase use cytoplasmic dynein motor complex for active transport along MT to reach the perinuclear space [55,56]. Dynein is composed of 12 to 14 different chains, and moves cargos at a velocity of ≈2–3.5 μm/s [57]. Cargo-dynein interactions are based on various binding partners including the dynein–dynactin complex [58]. Dynein-mediated transport is also used by protoparvovirus capsids to reach the NE [43,59,60,61,62,63,64,65,66,67,68], which is consistent with the velocity of 3.5 and ~2.0 μm/s observed for AAV2 and AAV9 [41,69,70].

## 3. Molecular Mechanisms of Nuclear Import

Nuclear viruses infecting non-dividing, e.g., resting cells must have developed strategies to pass the NE. The majority of them use NPC-mediated transport. An alternative pathway was described for the simian virus 40 (SV40), which not only traverses nuclear pores, but is also able to disrupt the inner nuclear membrane after internalization into the ER lumen [71].

NPCs are macromolecular structures composed of approximately 30 different protein species collectively called nucleoporins (Nups) [72,73]. Filaments extending in the cytoplasm are composed of, e.g., Nup358 (RanBP2) and Nup 214, whereas others such as Nup62 are part of NE-embedded scaffold rings, which form the hydrophobic transport channel [74]. On the nuclear side of NPC, eight filaments extrude the central part constituting the nuclear basket formed of Nups Tpr and Nup153. Nup153 may be flexible as its localization on the cytoplasmic side also has been reported [75]. The channel through NPC has a functional limit diameter of ~39 nm [76]. It is filled with a mesh of hydrophobic phenylalanine-glycine (FG) repeat-containing Nup domains [77,78,79] restricting size- and charge-dependent passive diffusion of molecules smaller than ~20–40 kDa (diameter < 4–5 nm) [80,81,82].

Active nuclear import of larger molecules of <25 megadaltons (diameter < 40 nm) is facilitated by nuclear import receptors (importins or karyopherins; Kaps) [83]. A total of nine different receptors have been described, differing in their cargo specificity. They mediate approximately 1000 translocations per NPC per second [84,85]. Given that there are 400 to 18,500 NPCs per nucleus [86], the transport receptors allow millions of exchange reactions per second per cell. The prerequisite for nuclear localization of molecular cargo is the NLSs. The classical NLSs (cNLSs) are formed of mono- or bipartite clusters of typically four to six basic amino acids. The prototype NLS found in SV40 T-antigen (SV40TAg) has the amino acid sequence PKKKRKV [87]. In the classical import pathway, the cNLS is bound by importin α (Kapα) adaptor protein, which binds to importin β (Kapβ1), and the formed trimeric importin α-importin β-NLS complex can enter the nucleus. Moreover, importin β can directly interact with cargos comprising an importin-β-binding domain [88].

Interaction of the import receptor with the NLS on the cargo can be regulated by post-translational modifications of the NLS or nearby residues. Alternatively, the NLS can be exposed or hidden, either based on structural changes of the protein with NLS (e.g., phosphorylation of SV40Tag) [89] or by protein–protein interactions (e.g., NFkB-IkB) [90]. Attachment of the cargo modifies the structure of import receptor, which allows its interaction with Nups and passage through the nuclear pore. The import reaction is terminated in the nuclear basket where the cargo-import receptor-complex binds to Nup153 [91]. This leads to dissociation of import receptor from its cargo, a process induced by GTP-bound form of Ras-related nuclear protein (Ran) [92,93]. While the cargo diffuses deeper into the nucleus, the import receptor-RanGTP-complex is exported through the NPC [88].

### 3.1. Nuclear Entry of Viral DNA Genomes

The nuclear import of most viruses and their genomes is restricted by the size of the virus capsid. Most viruses exceed the maximal size of the nuclear pore thus requiring their disassembly on the cytoplasmic side of NPC. This is well documented for adenoviral capsids, which disassemble to pentons and hexons after their docking to Nup214 and binding to kinesin 1 without need of nuclear import receptors [94,95]. It is assumed that the pulling forces by kinesin 1 on capsid bound to NPCs disintegrate them and lead to viral genome release [96,97]. The genome, in complex with the viral proteins VII, X and TP (terminal protein), then interacts indirectly with the nuclear import receptors. While, the identity of these import receptors has remained unclear, importin β, importin-7 and transportin-1 have been suggested together with adapter molecules hsp70 and/or histone H1 [95,98,99].

In contrast, herpes simplex virus capsids with a diameter of 125 nm associate with the NPCs either by direct binding of the inner tegument protein pUL25 to Nup214 or to importin β [100,101,102]. NPC-interaction acts as a triggering element for herpesviral DNA release, which is poorly understood but may involve proteolytic cleavage of UL36 [103]. During capsid assembly the HSV genome is packed in an energy-consuming process leading to a quasi-crystalline density [104]. Opening of the capsid at a site opposing the NPC causes an injection of DNA through the nuclear pore. After the repulsion comes to an end, the part of the genome which remains outside the nucleus and/or in the capsid is pulled into the nucleus by transcription of the nuclear genome end encoding for the immediate-early genes [105].

Very few viral capsids have diameters below the nuclear transport limit; amongst them parvoviral and circoviral capsids (15–30 nm), and that of hepatitis B virus (HBV; 36 nm). Nuclear import of circovirus genomes is practically uninvestigated, yet it was shown that the only capsid protein Cap comprises the NLS [106]. HBV capsids are composed of a single protein species, which contains the NLS [107] and also an importin-β-binding domain [108]. Accordingly, HBV capsids are imported into the nuclear basket using importin α and β, but in contrast to classical cargos the capsid shows high affinity for Nup153 so that the capsid becomes arrested inside the nuclear basket [109]. Genome release of HBV is poorly understood; however, it does require capsid disassembly to core protein dimers, as with adenoviruses.

### 3.2. Nuclear Import of Protoparvoviral Capsids

Acidification of parvoviral capsids upon endosomal entry leads to exposure of the N terminal part of VP1, hidden in the virion, and absent from VP2 (VP1 unique; VP1u). Aside of the PLA2 domain, described before, CPV VP1 comprises one stretch of basic amino acids (^4^PAKRARRGYK^13^) similar to classical NLSs (cNLSs) [110]. In addition, four stretches have been found within MVM VP1 [60]. This sequence was shown to mediate nuclear import of bovine serum albumin, and mutational analysis exhibited its importance in early CPV infection [111,112]. The closest relative of CPV, the feline panleukopeniavirus (FPV), as well as mink enterititis virus (MEV) and blue fox parvovirus Tai’an have similar sequences (^4^PAKRARRGLV^13^) in their VP1u proteins. Such cNLSs were further identified on *Ungulate parvovirus 1* species porcine parvovirus (PPV) VP1u and MVM VP1-N. In contrast, MVM, an example of the *Rodent protoparvovirus 1* species, have, in addition with two cNLSs on VP1 and VP2 proteins, a non-classical NLS (ncNLS) nuclear localization motif (NLM) in their C-terminal region of VP1/2. These NLMs are conserved amongst protoparvoviruses [22,60]. Other protoparvoviruses such as hamster parvovirus H1 and LuIII (*Rodent protoparvovirus 1*), and rat parvovirus 1 (*Rodent protoparvovirus 2*) have predicted NLS motifs (^4^PAKRAKRGWV^13^). The sequence analysis showed that such putative cNLS sequences are also found in VP1/VP2 of human protoparvoviruses, bufa-, tusa- and cutaviruses [113].

**Figure 1:**
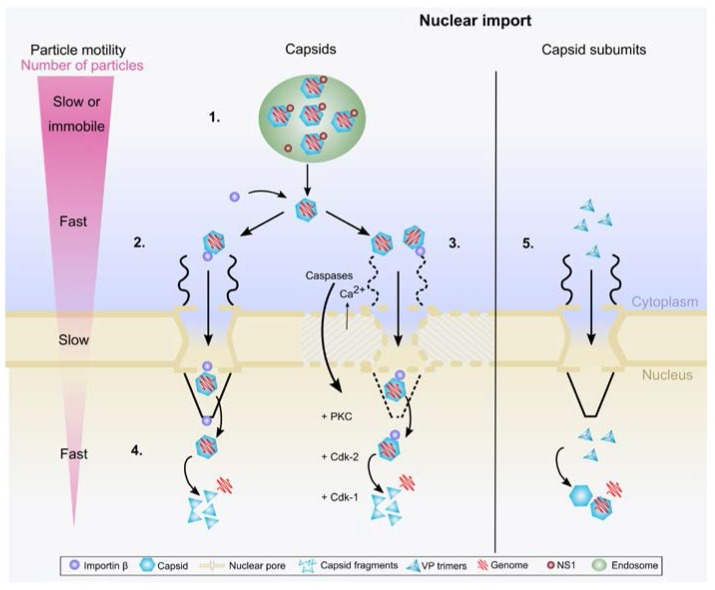
Overview on the parvoviral nuclear import. (**1**) Viruses enter the cell by endocytosis. Inside the endosome viral NLS is exposed and the NS1 protein is detached from the capsid. Endosomal escape is followed by cytoplasmic interaction of capsid with importin β. In the cytoplasm viruses exploit dynein- and microtubule-mediated transport towards the nucleus; (**2**) after reaching the nuclear envelope, viruses are transported through the nuclear pore complex; (**3**) the direct or importin-mediated indirect interaction with the nuclear pores may also induce nuclear envelope breakdown allowing virus entry into the nucleoplasm. Host cell caspases, nuclear inflow of Ca^2+^, and activation of mitotic enzymes including Cdk-1/2 and PKC are involved in this process; (**4**) nuclear import is followed by viral capsid disassembly and release of their genome in the nucleoplasm; (**5**) in the late stage of infection, the newly synthesized viral capsid proteins are imported into the nucleus as phosphorylated trimeric assembly intermediates.

Parvoviral capsids can be observed within the nucleus shortly after infection [62,114]. The observation of the conserved NLS combined with the attachment of at least importin β to cytosolic CPV capsids suggest that the capsids follow the classical nuclear import pathway. In fact, capsids have been observed on the nuclear side of the NPC [43]. Observations using the AAV2 and the protoparvovirus PV H-1 showed that their attachment to NE causes its local degradation [107]. This nuclear envelope breakdown (NEBD) is similar to mitotic NEBD, both of them need PKCα, cyclin-dependent kinases and Ca^2+^. The NEBD that is facilitated by host caspases and mitotic enzymes in PV H-1 and MVM infected cells [115,116,117]. Phospholipase A2 activity on MVM capsids has not been reported to be involved in causing NE disruption. Instead, the virus utilizes cellular caspases, especially caspase-3, a protease involved in NE breakdown during apoptosis, to facilitate nuclear membrane disruptions [117]. Interestingly, piercing or bending of the NE by MT during mitosis was shown to trigger NEBD. The capsid attachment to Nup153 suggests that capsids are able to enter the nuclear basket allowing for their access and permeabilization of the NE. This process is independent on PLA2, at least for AAV2 [118]. Of note, Ca^2+^ release was a prerequisite for parvovirus-mediated activation of PKC, as of cyclin-dependent kinases, yet called for parvovirus interaction with the NPC (e.g., Nup358, Nup62 and Nup153). Finally, microinjection of MVM capsids into the cytoplasm of *Xenopus laevis* oocytes resulted in the damage of nuclear envelope allowing NPC-independent entry of capsids [116]. Notwithstanding the apparent contradiction (Figure 1), the observations in concert suggest that protoparvoviruses first attach to the NPC directly or via importin β (and potentially importin α), and then penetrate the nuclear pore. The nuclear import through the NPC is able to trigger an extensive modification of nuclear envelope leading to NEBD facilitating the passage of capsid into the nucleus, however, this does not rule out the possibility that a portion of the capsids might be actively transported into the nucleus by importins through the intact NPCs and the nuclear envelope. At the moment, part of underlying mechanisms and specific interactions leading to parvovirus nuclear entry are still unknown and additional studies are required for better understanding these processes.

### 3.3. Nuclear Entry of Capsid Subunits

Although the importance of the conventional NLS on parvoviral VP1 is substantiated by its phylogenetic conservation, its activities during the viral life cycle are not fully understood. It is known to be involved in NPC interaction during viral entry and in nuclear import of newly synthesized viral capsid proteins during the S-phase of the cell cycle (Figure 1). Nuclear translocation of the newly formed VP2 protein, without cNLS, is driven by a structured nuclear localization motif (NLM) [119]. The studies with MVM have demonstrated that VP1 and VP2 form a trimer prior to nuclear entry. It seems that the NLMs dominate over the NLSs of VP1, driving VP2 and VP1/VP2 subunits (2VP2/1VP1, and 3VP2) into the nucleus. The trimers have the capacity to form capsids in the cytosol when subjected to cell contact-triggered density-arrest signals. This has been shown in MVM studies, where the cell cycle arrest in G0, G1 or G1/S transition inhibits nuclear transport of the VPs [119]. It is also known that the NLM is exposed on VP1/VP2 trimers but not on assembled capsids [22,60]. VP1/VP2 heterotrimers undergo modifications that are required for their nuclear import in a cell cycle-dependent manner. Searching for the molecular mechanism revealed that VP2 phosphorylation by the protein kinase Raf-1 is required [120]. Accordingly, VP1/VP2 heterotrimers expressed in insect cells, which are devoid of Raf-1, failed to enter the nucleus and Raf-1 expression correlated with cell permissiveness to MVM infection [120].

## 4. End of Import-Capsid Disassembly in the Nucleus

Viral gene expression in the nucleus requires uncoating and genome delivery from a protecting protein shell. As protoparvovirus capsids enter the nucleus interior, they must maintain their genome in an encapsidated state until they reach in the nucleoplasm. How and where the capsids uncoat for gene expression not until nuclear entry is a long-standing question remaining to be answered. Earlier studies have shown that in infectious parvovirus particles, 20–30 nucleotides of the 5’ end are exposed on the capsid surface [5,16,121,122]. However, the roles of these 5’ ends or of the NLS in nuclear release of viral DNA are unknown. Although the molecular mechanisms of capsid disassembly are yet unknown, recent studies of intranuclear CPV capsids dynamics have indicated that slow moving nucleoplasmic CPV capsids are accompanied by the rapidly moving capsid-derived components, either remnants thereof or dissociated constituent proteins. This shows that capsids are disassembled after their entry into the nucleoplasm [43]. Within the nucleus, once the genome becomes released, transcription and replication are initiated via nuclear host proteins. Recent studies have shown that epigenetic modification of the parvoviral genome, specifically the activation of promoters, plays a critical role in progression of the parvoviral life cycle [123,124,125,126].

## 5. Concluding Remarks

The nuclear pore complex constitutes a gate for the entry of viral DNA genome into the nucleus. Viruses have found multiple strategies to allow the passage of their genome. They include disassembly either in the cytosol or at the NPC followed by nuclear import of a genomic complex through the NPC. The genome of some viruses can be injected into the nucleus from a capsid docked at the pore complex. Parvoviruses with capsid diameters falling below the exclusion limit fit in through the NPC. The import is facilitated by NLSs and interactions with nuclear import machinery. Subsequently, these interactions can promote viral capsid entry by inducing NE disintegration. The elucidation of the diverse strategies of parvoviral nuclear import will no doubt shed new light on the complexity of this process and lead to improved means of cancer treatment and gene therapy.
